# Association of *RAN* and *RANBP2* Gene Polymorphisms With Glioma Susceptibility in Chinese Children

**DOI:** 10.1002/cnr2.2136

**Published:** 2024-07-23

**Authors:** Qianru Lin, Wei Chen, Jiating Tan, Sifan Qian, Huarong Su, Liang Zhao, Li Yuan, Jichen Ruan, Xiaokai Huang, Haixia Zhou

**Affiliations:** ^1^ Department of Hematology The Second Affiliated Hospital and Yuying Children's Hospital of Wenzhou Medical University Wenzhou Zhejiang China; ^2^ Department of Pediatric Surgery, Guangzhou Women and Children's Medical Center, Guangdong Provincial Clinical Research Center for Child Health Guangzhou Medical University Guangzhou Guangdong China; ^3^ Department of Pathology, Guangzhou Women and Children's Medical Center Guangzhou Medical University Guangzhou Guangdong China; ^4^ The Key Laboratory of Pediatric Hematology and Oncology Diseases of Wenzhou The Second Affiliated Hospital and Yuying Children's Hospital of Wenzhou Medical University Wenzhou Zhejiang China

**Keywords:** glioma, polymorphism, *RAN*, *RANBP2*, susceptibility

## Abstract

**Background:**

Glioma is the most prevalent pediatric central nervous system malignancy. RAN, member RAS oncogene family (*RAN*), is a key signaling molecule that regulates the polymerization of microtubules during mitosis. RAN binding protein 2 (*RANBP2*) is involved in DNA replication, mitosis, metabolism, and tumorigenesis. The effects of *RAN* and *RANBP2* gene polymorphisms on glioma susceptibility in Chinese children are currently unknown.

**Aims:**

This study aimed to evaluate the association between *RAN* and *RANBP2* gene polymorphisms and glioma susceptibility in Chinese children.

**Methods and Results:**

We recruited 191 patients with glioma and 248 children without cancer for this case–control study. Polymerase chain reaction‐based TaqMan was applied to gene sequencing and typing. Logistic regression model‐calculated odds ratio and 95% confidence interval were used to verify whether the gene polymorphisms (*RAN* rs56109543 C>T, rs7132224 A>G, rs14035 C>T, and *RANBP2* rs2462788 C>T) influence glioma susceptibility. Based on age, gender, tumor subtype, and clinical stage, stratified analyses of risk and protective genotypes were conducted. *p* values for mutant genotype analyses were all >0.05, indicating no significant correlation between these gene polymorphisms and glioma risk.

**Conclusion:**

*RAN* and *RANBP2* gene polymorphisms were not found to be statistically significantly associated with glioma susceptibility in Chinese children. Other potential functional gene polymorphism loci of *RAN* and *RANBP2* will need to be evaluated in the search for novel glioma biomarkers.

**Abbreviations**
AOR: adjusted odds ratio
CI: confidence interval
CNS: central nervous system
FTO: fat mass and obesity‐associated protein
GBM: glioblastoma
HWE: Hardy–Weinberg equilibrium
IDH: isocitrate dehydrogenase
OR: odds ratio
*RAN*
: RAN, member RAS oncogene family
*RANBP2*
: RAN binding protein 2
RT‐PCR: real‐time polymerase chain reaction
SNP: single nucleotide polymorphism
TF: transcription factor
WHO: World Health Organization

## 1 Introduction

Glioma is a solid primary brain tumor derived from neuroglial stem cells or progenitor cells. It is the most prevalent pediatric central nervous system (CNS) cancer [1]. Malignant brain tumor incidence in children is increasing in the United States, with an annual increase that ranged from 0.5% to 0.7% during 2008 and 2017 [2, 3]. Glioma prevalence varies based on age, sex, and ethnic background [4]. Although most pediatric gliomas are benign, survival is poor in specific tumor subtypes. Although most pediatric gliomas are benign, survival is poor in specific tumor subtypes. The five‐year relative survival rate for glioblastoma (GBM) in patients aged 0–14 years is around 19.9% [1]. Therefore, early and accurate diagnosis of glioma is crucial for improving prognosis. In recent decades, genetic analysis has become a highly discussed topic due to its potential to aid in glioma diagnosis, classification, prognosis, and treatment response.

According to the 2021 World Health Organization (WHO) classification of CNS tumors, histological performance and molecular characteristics play critical roles in the new glioma classification criteria [5]. Glioma development is often accompanied by molecular mutations such as *IDH* mutation, *TERT* promoter mutations, *1p/19q* codeletion, and *RELA* fusions [5, 6, 7, 8, 9, 10]. Schönrock et al. reported that *MEOX2* is a carcinogenic transcriptional regulator in GBM [11]. Le Boiteux et al. conducted a molecular analysis of glioma stem cells from 70 glioma samples and found that *H3K27me3* loss caused *HOX* gene upregulation in glioma cells [12]. *METTL3* mediates m^6^A modification, accelerating attenuation of the *UBXN1* mRNA in glioma and promoting carcinogenesis [13, 14]. Molecular diagnostic markers have become a fundamental conceptual foundation regarding glioma molecular pathogenesis. Some studies have verified that variations in specific single nucleotide polymorphisms (SNPs) significantly increase the risk of glioma [15, 16, 17, 18]. These findings provide a reasonable basis for predicting the risk and survival rate of gliomas. Studies have found distinct variations in the biomarkers of adult and pediatric gliomas. For instance, biomarkers such as *PTEN* deletion, *IDH* mutations, or *EGFR* amplification are common in adult patients yet rare in pediatric patients [19, 20, 21]. Therefore, adult glioma findings should not be assumed to generalize to pediatric glioma. Investigating glioma biomarkers in various age groups is crucial, which will improve the diagnostic methodologies and offer more precise treatment alternatives to glioma patients. Nevertheless, there remain numerous limitations to our understanding of how gene polymorphisms relate to pediatric glioma susceptibility.

RAN, member RAS oncogene family (*RAN*), encodes a small GTP‐binding protein that combines GTP or GDP and circulates between these states, forming the RAN cycle. The RAN cycle, regulated by RAN regulatory proteins, controls the directionality of nucleocytoplasmic transport [22, 23, 24]. RAN protein regulates microtubule polymerization and spindle assembly during mitosis. Its mutation could hinder DNA synthesis [25]. Some studies have found *RAN* overexpression in the cells of several tumors, including neuroblastoma [26], breast cancer [27, 28], pancreatic cancer [23], gastric cancer [29], and squamous cell carcinoma of the head and neck [30], indicating that it may influence cancer susceptibility. Knockdown of the *RAN* gene reduces expression of c‐Met and its downstream signaling target ERK1/2, significantly reducing the ability of A375 and G361 melanoma cells to migrate and invade [31]. *RAN* rs7132224 A>G was found to increase Wilms tumor risk [32]. RAN binding protein 2 (*RANBP2*) forms the nuclear pore complex and regulates the transport of proteins and other molecules into or out of the nucleus. It also participates in DNA replication and mitosis [33]. As reported in a study of hepatocarcinogenesis, *RANBP2* may cause glycosylation of fat mass and obesity‐associated protein (FTO) and promote the development of hepatocellular carcinoma [34]. However, the associations of *RAN* and *RANBP2* gene polymorphisms with glioma risk have not been examined.

The research herein assessed the associations of *RAN* and *RANBP2* gene polymorphisms with glioma susceptibility among 439 Chinese children.

## 2 Materials and Methods

### 2.1 Study Participants

The retrospective research evaluated data from 439 children, including 191 patients with glioma and 248 controls without cancer (Table 1). The study selected patients according to the following inclusion criteria: (1) children or adolescents aged 0–18 years, (2) the Han nationality, (3) patients definitively diagnosed with glioma based on histopathology, and (4) patients treated in the designated hospitals (the Second Affiliated Hospital and Yuying Children's Hospital of Wenzhou Medical University and the Guangzhou Women and Children's Medical Center). Children with concomitant or suspected co‐occurrence of other tumors were excluded. The control group was randomly selected from the contemporaries who underwent routine physical examinations in the designated hospitals during the same period. All of the controls denied any tumor history or tumor‐related family history. The study classified participants by age (<60 and ≥60 months) and gender (female and male). The classification and staging of tumors were based on WHO criteria. Data that were not available were noted as “NA.” The ethics committees of the Second Affiliated Hospital and Yuying Children's Hospital of Wenzhou Medical University and the Guangzhou Women and Children's Medical Center authorized the research. All participants' legal guardians completed informed consent.

**TABLE 1:** Frequency distribution of selected variables in glioma patients and cancer‐free controls.

	Cases (*N* = 191)		Controls (*N* = 248)		
Variables	No.	%	No.	%	*p* ^a^
Age range, month	2.60–168.00		4.00–168.00		0.997
Mean ± SD	62.74 ± 47.28		53.90 ± 33.47		
<60	97	50.79	126	50.81	
≥60	94	49.21	122	49.19	
Gender					0.329
Female	89	46.60	104	41.94	
Male	102	53.40	144	58.06	
Subtypes					
Astrocytic tumors	136	71.20	/	/	
Ependymoma	33	17.28	/	/	
Neuronal and mixed neuronal‐glial tumors	14	7.33	/	/	
Embryonal tumors	7	3.66	/	/	
NA	1	0.52	/	/	
WHO stages					
I	110	57.59	/	/	
II	38	19.90	/	/	
III	17	8.90	/	/	
IV	25	13.09	/	/	
NA	1	0.52	/	/	

Abbreviations: NA, not available; SD, standard deviation.

^a^ Two‐sided *χ* ^2^ test for distributions between glioma patients and cancer‐free controls.

### 2.2 Polymorphism Selections and Genotyping

We investigated the SNPs of *RAN* and *RANBP2* from the Single Nucleotide Polymorphism Database (dbSNP) (http://www.ncbi.nlm.nih.gov/). The biological function of SNPs was forecasted by SNPinfo (http://snpinfo.niehs.nih.gov/). The likely functional SNPs (*RAN* rs56109543 C>T, rs7132224 A>G, rs14035 C>T, and *RANBP2* rs2462788 C>T) were selected. The genomic DNA was extracted from human peripheral blood samples using the TIANamp blood DNA kit (TIANGEN BIOTECH, Beijing). Subsequently, genotyping was carried out using the Applied Biosystems 7900HT Sequence Detection System. This system performs TaqMan probe‐based real‐time polymerase chain reaction (RT‐PCR) to sequence and genotype the genetic material. The TIANtough Genotyping qPCR PreMix (Probe) (TIANGEN BIOTECH, Beijing) was utilized for this process. The RT‐PCR program consisted of an initial denaturation step at 95°C for 2 min, followed by 40 cycles of denaturation at 95°C for 15 s and annealing/extension at 60°C for 20 s.

### 2.3 Statistical Analysis

We used the *χ* ^2^ tests with two‐sided significance testing to examine genotype distributions between patients and controls. The goodness‐of‐fit test estimated the Hardy–Weinberg equilibri.um (HWE). We conducted univariate and multivariate logistic regression analyses. Odds ratios (ORs) and 95% confidence intervals (CIs) were used to assess the relation between the SNPs and risk of developing glioma. Moreover, the data were adjusted according to age and gender, and the adjusted odds ratios (AOR) and 95% CIs were calculated for stratified analysis. We recognized a difference as statistically significant when *p* < 0.05.

## 3 Results

### 3.1 Participant Characteristics

A summary of the frequency distributions for selected participant data is shown in Table 1. Frequency matching between the case and control groups meant that they did not differ significantly on age (*p* = 0.997) or sex (*p* = 0.329). The four tumor subtypes in the case group were astrocytic tumors (*n* = 136, 71.20%); ependymoma (*n* = 33, 17.28%); neuronal and mixed neuronal glial tumors (*n* = 14, 7.33%); and embryonal tumors (*n* = 7, 3.66%). According to the WHO grading standard, patients were classified as I (*n* = 110, 57.59%), II (*n* = 38, 19.90%), III (*n* = 17, 8.90%), and IV (*n* = 25, 13.09%). Clinical data were unavailable for one patient, whom we could thus not classify by subtype or WHO stage.

### 3.2 Relations Between *RAN* and *RANBP2* SNPs and Glioma Susceptibility

Four *RAN* and *RANBP2* gene SNPs were analyzed (*RAN* rs56109543 C>T, rs7132224 A>G, rs14035 C>T, and *RANBP2* rs2462788 C>T). Table 2 displays all genotype data for cases and controls. HWE test results for the SNPs (HWE = 0.447 for rs56109543 C>T, HWE = 0.799 for rs7132224 A>G, HWE = 0.995 for rs14035 C>T, and HWE = 0.465 for rs2462788 C>T) indicated that the samples were representative. All genotype *p* values were >0.05, indicating that none differed statistically significantly. Despite this, we defined risk genotypes as carriers with rs56109543 CT/TT (adjusted OR = 1.36, 95% CI = 0.93–1.93, *p* = 0.150), rs7132224 AG/GG (adjusted OR = 1.20, 95% CI = 0.82–1.76, *p* = 0.344), and rs14035 CT/TT (adjusted OR = 1.26, 95% CI = 0.85–1.88, *p* = 0.253). The TT genotype of rs56109543 had the highest OR value (OR = 2.06, 95% CI = 0.58–7.31, *p* = 0.266). The lowest OR value was for the CT genotype of rs2462788 (OR = 0.94, 95% CI = 0.48–1.84, *p* = 0.856).

**TABLE 2:** *RAN* and *RANBP2* gene polymorphisms with glioma susceptibility in Chinese children.

Genotype	Cases (*N* = 191)	Controls (*N* = 248)	*p* ^a^	Crude OR (95% CI)	*p*	Adjusted OR (95% CI)^b^	*p* ^b^
*RAN* rs56109543 C>T (HWE = 0.447)							
CC	126 (65.97)	178 (71.77)		1.00		1.00	
CT	58 (30.37)	66 (26.61)		1.24 (0.82–1.89)	0.313	1.30 (0.85–1.99)	0.221
TT	7 (3.66)	4 (1.61)		2.47 (0.71–8.62)	0.156	2.06 (0.58–7.31)	0.266
Additive			0.118	1.33 (0.93–1.91)	0.119	1.34 (0.93–1.93)	0.115
Dominant	65 (34.03)	70 (28.23)	0.191	1.31 (0.87–1.97)	0.192	1.36 (0.90–2.04)	0.150
Recessive	184 (96.34)	244 (98.39)	0.173	2.32 (0.67–8.05)	0.185	1.92 (0.54–6.79)	0.312
*RAN* rs7132224 A>G (HWE = 0.799)							
AA	101 (52.88)	141 (56.85)		1.00		1.00	
AG	78 (40.84)	91 (36.69)		1.20 (0.81–1.78)	0.374	1.23 (0.83–1.84)	0.305
GG	12 (6.28)	16 (6.45)		1.05 (0.48–2.31)	0.909	1.04 (0.47–2.31)	0.923
Additive			0.520	1.11 (0.81–1.50)	0.520	1.12 (0.82–1.53)	0.472
Dominant	90 (47.12)	107 (43.15)	0.406	1.17 (0.80–1.72)	0.407	1.20 (0.82–1.76)	0.344
Recessive	179 (93.72)	232 (93.55)	0.943	0.97 (0.45–2.11)	0.943	0.95 (0.44–2.08)	0.906
*RAN* rs14035 C>T (HWE = 0.995)							
CC	120 (62.83)	167 (67.34)		1.00		1.00	
CT	64 (33.51)	73 (29.44)		1.22 (0.81–1.84)	0.341	1.27 (0.84–1.92)	0.262
TT	7 (3.66)	8 (3.23)		1.22 (0.43–3.45)	0.711	1.22 (0.43–3.51)	0.710
Additive			0.352	1.18 (0.84–1.65)	0.352	1.21 (0.85–1.70)	0.288
Dominant	71 (37.17)	81 (32.66)	0.325	1.22 (0.82–1.81)	0.325	1.26 (0.85–1.88)	0.253
Recessive	184 (96.34)	240 (96.77)	0.802	1.14 (0.41–3.21)	0.801	1.13 (0.40–3.22)	0.816
*RANBP2* rs2462788 C>T (HWE = 0.465)							
CC	175 (91.62)	226 (91.13)		1.00		1.00	
CT	16 (8.38)	22 (8.87)		0.94 (0.48–1.84)	0.856	0.94 (0.48–1.84)	0.856
TT	0 (0.00)	0 (0.00)		/	/	/	/
Additive			0.855	0.94 (0.48–1.84)	0.856	0.94 (0.48–1.84)	0.846
Dominant	16 (8.38)	222 (8.87)	0.855	0.94 (0.48–1.84)	0.856	0.94 (0.48–1.84)	0.856
Combined effect of risk genotypes^c^							
0	97 (50.79)	141 (56.85)	0.266	1.00		1.00	
1	24 (12.57)	26 (10.48)		1.34 (0.73–2.48)	0.347	1.35 (0.73–2.50)	0.346
2	8 (4.19)	11 (4.44)		1.06 (0.41–2.73)	0.908	1.11 (0.43–2.87)	0.833
3	62 (32.46)	70 (28.23)		1.29 (0.84–1.98)	0.248	1.33 (0.86–2.05)	0.196
0	97 (50.79)	141 (56.85)		1.00		1.00	
1–3	94 (49.21)	107 (43.15)	0.206	1.28 (0.87–1.87)	0.206	1.31 (0.89–1.92)	0.165

Abbreviations: CI, confidence interval; HWE, Hardy–Weinberg equilibrium; OR, odds ratio.

^a^ *χ* ^2^ test for genotype distributions between glioma patients and cancer‐free controls.

^b^ Adjusted for age and gender.

^c^ Risk genotypes were carriers with rs56109543 CT/TT, rs7132224 AG/GG, and rs14035 CT/TT genotypes.

### 3.3 Stratification Analysis

Table 3 displays the results of the stratification analysis of risk genotypes for glioma susceptibility. The rs56109543 mutation appeared to have no significant correlation with glioma risk in individuals, regardless of whether they were younger than 60 months or older. There were no significant differences observed in genders, tumor subtypes, or clinical stages. Although the rs56109543 mutation might have acted as a protective factor for patients with clinical stage II glioma, this association was not statistically significant (OR = 0.79, 95% CI = 0.36–1.76, *p* = 0.570). Cases who carried 1–3 risk genotypes (including rs56109543 CT/TT, rs7132224 AG/GG, and rs14035 CT/TT) showed no significant difference in glioma risk compared with controls when considering factors such as ages, genders, subtypes, or clinical stages. These results suggested that these genotypes might not link to the risk of glioma.

**TABLE 3:** Stratification analysis of risk genotypes with glioma susceptibility.

	rs56109543 (cases/controls)		AOR^a^		Risk genotypes^b^ (cases/controls)		AOR^a^	
Variables	CC	CT/TT	(95% CI)	*p* ^a^	0	1–3	(95% CI)	*p* ^a^
Age, month								
<60	60/89	37/37	1.51 (0.86–2.65)	0.154	45/70	52/56	1.45 (0.85–2.47)	0.169
≥60	66/89	28/33	1.14 (0.63–2.07)	0.670	52/71	42/51	1.12 (0.65–1.93)	0.683
Gender								
Females	62/74	27/30	1.09 (0.58–2.03)	0.795	44/61	45/43	1.47 (0.83–2.61)	0.189
Males	64/104	38/40	1.60 (0.93–2.77)	0.092	53/80	49/64	1.20 (0.72–2.00)	0.494
Subtypes								
Astrocytic tumors	88/178	48/70	1.42 (0.90–2.25)	0.129	69/141	67/107	1.29 (0.84–1.97)	0.251
Ependymoma	23/178	10/70	1.06 (0.48–2.37)	0.880	15/141	18/107	1.55 (0.74–3.23)	0.248
Neuronal and mixed neuronal‐glial tumors	10/178	4/70	0.97 (0.29–3.22)	0.957	8/141	6/107	0.93 (0.31–2.80)	0.900
Embryonal tumors	4/178	3/70	3.95 (0.65–24.02)	0.136	4/141	3/107	1.23 (0.24–6.30)	0.807
Clinical stages								
I	69/178	41/70	1.55 (0.96–2.50)	0.074	54/141	56/107	1.38 (0.88–2.18)	0.162
II	29/178	9/70	0.79 (0.36–1.76)	0.570	21/141	17/107	1.07 (0.54–2.13)	0.845
III	11/178	6/70	1.36 (0.48–3.84)	0.563	7/141	10/107	1.89 (0.69–5.16)	0.215
IV	16/178	9/70	1.66 (0.67–4.13)	0.278	14/141	11/107	1.12 (0.47–2.68)	0.806
I + II	98/178	50/70	1.33 (0.85–2.07)	0.208	75/141	73/107	1.30 (0.86–1.96)	0.216
III + IV	27/178	15/70	1.48 (0.73–2.98)	0.274	21/141	21/107	1.38 (0.71–2.69)	0.339

Abbreviations: AOR, adjusted odds ratio; CI, confidence interval; OR, odds ratio.

^a^ Adjusted for age and gender, omitting the corresponding stratify factor.

^b^ Risk genotypes were carriers with rs56109543 CT/TT, rs7132224 AG/GG, and rs14035 CT/TT genotypes.

Similarly, we investigated the association between the *RANBP2* rs2462788 C>T polymorphism and glioma risk (Table 4). For individuals aged less than 60 months, there was no significant association between *RANBP2* rs2462788 C>T polymorphism and glioma risk (AOR = 0.90, 95% CI = 0.33–2.45, *p* ^a^ = 0.832). Similarly, for individuals aged 60 months or older, there is also no significant association (AOR = 0.98, 95% CI = 0.40–2.45, *p* ^a^ = 0.971).

**TABLE 4:** Stratification analysis between *RANBP2* rs2462788 C>T polymorphism and glioma risk.

	rs2462788 (cases/controls)		Crude OR		AOR^a^	
Variables	CC	CT/TT	(95% CI)	*p*	(95% CI)	*p* ^a^
Age, month						
<60	97/116	7/10	0.90 (0.33–2.46)	0.841	0.90 (0.33–2.45)	0.832
≥60	85/110	9/12	0.97 (0.39–2.41)	0.949	0.98 (0.40–2.45)	0.971
Gender						
Females	83/94	6/10	0.68 (0.24–1.95)	0.473	0.69 (0.24–1.99)	0.493
Males	92/132	10/12	1.20 (0.50–2.88)	0.691	1.17 (0.48–2.83)	0.733
Subtype						
Astrocytic tumors	124/226	12/22	0.99 (0.48–2.08)	0.988	1.01 (0.48–2.12)	0.981
Ependymoma	31/226	2/22	0.66 (0.15–2.96)	0.590	0.74 (0.16–3.37)	0.699
Neuronal and mixed neuronal‐glial tumors	12/226	2/22	1.71 (0.36–8.15)	0.499	1.92 (0.39–9.38)	0.421
Embryonal tumors	7/226	0/22	/	/	/	/
Clinical stages						
I	100/226	10/22	1.03 (0.47–2.25)	0.946	1.04 (0.47–2.28)	0.932
II	38/226	0/22	/	/	/	/
III	14/226	3/22	2.20 (0.59–8.25)	0.242	2.60 (0.67–10.06)	0.167
IV	22/226	3/22	1.40 (0.39–5.06)	0.607	1.46 (0.39–5.51)	0.576
I + II	138/226	10/22	0.74 (0.34–1.62)	0.457	0.74 (0.34–1.62)	0.456
III + IV	36/226	6/22	1.71 (0.65–4.51)	0.277	1.64 (0.62–4.37)	0.320

Abbreviations: AOR, adjusted odds ratio; CI, confidence interval; OR, odds ratio.

^a^ Adjusted for age and gender, omitting the corresponding stratify factor.

Females carrying the *RANBP2* rs2462788 C>T polymorphism mutation appeared to have a reduced risk of developing gliomas (AOR = 0.69). However, males with the same mutation seemed to face a higher risk of developing gliomas (AOR = 1.17), although the differences were not statistically significant (*p* ^a^ of females = 0.493, *p* ^a^ of males = 0.733). There was no significant difference in risk among different types of gliomas. In terms of tumor classification and staging, no significant association was found between carrying the mutation and the risk of glioma.

## 4 Discussion

To investigate the relations between *RAN*/*RANBP2* gene polymorphisms and glioma risk, we conducted a multicenter clinical study. Herein, no association of either *RAN* or *RANBP2* gene polymorphism was found in relation to Chinese children's glioma susceptibility. Although our results did not support the association of the selected SNPs with glioma, the association of additional *RAN* and *RANBP2* SNPs with glioma remains worthy of further investigation.

*RAN* is one of the most often mentioned genes regarding its function in tumorigenesis. Strongly expressed *RAN* was found in GBM tissues [35]. Several RAN‐binding proteins have been found to be involved in glioma development. Hou et al. revealed that downregulation of *RANBP10* inhibits important GBM cellular activities, including cell proliferation, migration, invasion, and tumor growth [36]. The focal deletion of *RANBP6* focal deletion in GBM was found by Oldrini et al. [37] Recently, *RANBP1* expression was found to be elevated in glioma stem cells, and linked to poor prognosis through its regulation of the cytokine IL‐18 [38].

Despite the potential significance of the relations between *RAN* and glioma, only a few studies have investigated this topic. Ayanlaja et al. were the first to report that the RAN signaling pathway regulates *DCX* expression in the nucleus of glioma cells, contributed to glioma progression [39]. According to Huang et al., *RAN* is activated by chromatin‐bound RCC1, promoting GBM development [40]. In experiments by Woo, *RAN* partially blocked the apoptosis induced by paclitaxel in U373MG cells, a human GBM‐derived cell line [41]. Schmits et al. discovered an antigen in the serum of astrocytoma patients, an expression product of *RANBP2*. However, antibodies against this antigen were comparable between glioma patients and normal controls [42]. These findings imply that the expression of *RAN* and *RANBP2* could play a role in glioma development and progression. The underlying mechanisms of influence require further research. Consequently, despite the lack of evidence linking the selected SNPs to glioma risk, the study of additional potential SNPs remains a crucial next step.

To our knowledge, ours is the first group in China to investigate the susceptibility of *RAN* and *RANBP2* gene polymorphisms in pediatric glioma risk. Despite the results' lack of support for the influence of the four SNPs on glioma susceptibility, other aspects need to be examined. For instance, the quality of life among patients with glioma should be taken into consideration. Additionally, the *RAN* and *RANBP2* genes may have other functional polymorphisms that should be explored. To this end, it has been well documented that various transcription factors (TFs) are transported into the nucleus in a *RAN*‐dependent manner, and several of these TFs impact cellular proliferation and differentiation. Importantly, the translocation of signal transducer and activator of transcription 3 (*STAT3*) requires *RAN* to subsequently stimulate cellular differentiation, proliferation, and tumor cell invasion [43]. Given that constitutive activation of *STAT3* in glioma cells has been shown to orchestrate immune evasion and exert a pivotal role in shaping tumor immune microenvironment‐promoting immunosuppression and resistance to glioma immunotherapy [44, 45], aberrations in *RAN* expression and other functional SNPs of *RAN* affecting *STAT3* in glioma should be evaluated as potential biomarkers. Finally, due to the small sample size, larger samples and analysis from multicenter studies are needed to help confirm our conclusions.

In summary, while we did not find a strong link between *RAN* and *RANBP2* gene polymorphisms and glioma susceptibility in Chinese children, this study did help advance glioma genetics research.

## 5 Conclusions

No evidence was found to support the role of any of the four selected SNPs in significantly affecting the risk of glioma among Chinese children. Therefore, it is now necessary to further explore other potential functional gene polymorphism loci of *RAN* and *RANBP2* to find novel glioma biomarkers.

## Author Contributions

**Qianru Lin:** conceptualization, formal analysis, writing – original draft. **Wei Chen:** investigation, formal analysis, data curation. **Jiating Tan:** methodology. **Sifan Qian:** methodology. **Huarong Su:** methodology. **Liang Zhao:** validation. **Li Yuan:** writing – review and editing. **Jichen Ruan:** resources, project administration, funding acquisition. **Xiaokai Huang:** data curation, writing – review and editing. **Haixia Zhou:** conceptualization, resources, supervision, project administration.

## Ethics Statement

The ethics committees of the Second Affiliated Hospital and Yuying Children's Hospital of Wenzhou Medical University and the Guangzhou Women and Children's Medical Center authorized the research. All participants' legal guardians completed informed consent.

## Conflicts of Interest

The authors declare no conflicts of interest.

## Data Availability

The data pertaining to this study are within the published article. Any additional data will be made available by the corresponding author on reasonable request.

## References

[1] Q. T. Ostrom , M. Price , C. Neff , et al., “CBTRUS Statistical Report: Primary Brain and Other Central Nervous System Tumors Diagnosed in the United States in 2015‐2019,” Neuro‐Oncology 24, no. Suppl 5 (2022): v1–v95. 36196752 10.1093/neuonc/noac202 PMC9533228

[2] M. Adel Fahmideh and M. E. Scheurer , “Pediatric Brain Tumors: Descriptive Epidemiology, Risk Factors, and Future Directions,” Cancer Epidemiology, Biomarkers & Prevention 30, no. 5 (2021): 813–821. 10.1158/1055-9965.EPI-20-1443 33653816

[3] K. D. Miller , Q. T. Ostrom , C. Kruchko , et al., “Brain and Other Central Nervous System Tumor Statistics, 2021,” CA: A Cancer Journal for Clinicians 71, no. 5 (2021): 381–406. 34427324 10.3322/caac.21693

[4] N. Patil , M. E. Kelly , D. N. Yeboa , et al., “Epidemiology of Brainstem High‐Grade Gliomas in Children and Adolescents in the United States, 2000‐2017,” Neuro‐Oncology 23, no. 6 (2021): 990–998. 33346835 10.1093/neuonc/noaa295 PMC8168816

[5] D. N. Louis , A. Perry , P. Wesseling , et al., “The 2021 WHO Classification of Tumors of the Central Nervous System: A Summary,” Neuro‐Oncology 23, no. 8 (2021): 1231–1251. 34185076 10.1093/neuonc/noab106 PMC8328013

[6] S. Han , Y. Liu , S. J. Cai , et al., “IDH Mutation in Glioma: Molecular Mechanisms and Potential Therapeutic Targets,” British Journal of Cancer 122, no. 11 (2020): 1580–1589. 32291392 10.1038/s41416-020-0814-x PMC7250901

[7] J. J. Miller , “Targeting IDH‐Mutant Glioma,” Neurotherapeutics 19, no. 6 (2022): 1724–1732. 35476295 10.1007/s13311-022-01238-3 PMC9723039

[8] S. Ohba , K. Kuwahara , S. Yamada , M. Abe , and Y. Hirose , “Correlation Between IDH, ATRX, and TERT Promoter Mutations in Glioma,” Brain Tumor Pathology 37, no. 2 (2020): 33–40. 32227259 10.1007/s10014-020-00360-4

[9] A. McAleenan , H. E. Jones , A. Kernohan , et al., “Diagnostic Test Accuracy and Cost‐Effectiveness of Tests for Codeletion of Chromosomal Arms 1p and 19q in People With Glioma,” Cochrane Database of Systematic Reviews 3, no. 3 (2022): CD013387. 35233774 10.1002/14651858.CD013387.pub2 PMC8889390

[10] R. Wang , Q. Li , X. Chu , N. Li , H. Liang , and F. He , “LncBIRC3‐OT Promotes the Malignant Progression of Glioma by Interacting With RELA to Upregulate Stanniocalcin‐1 Expression,” Heliyon 9, no. 11 (2023): e21777. 38034675 10.1016/j.heliyon.2023.e21777 PMC10681922

[11] A. Schönrock , E. Heinzelmann , B. Steffl , et al., “MEOX2 Homeobox Gene Promotes Growth of Malignant Gliomas,” Neuro‐Oncology 24, no. 11 (2022): 1911–1924. 35468210 10.1093/neuonc/noac110 PMC9629421

[12] E. Le Boiteux , F. Court , P.‐O. Guichet , et al., “Widespread Overexpression From the Four DNA Hypermethylated HOX Clusters in Aggressive (IDHwt) Glioma Is Associated With H3K27me3 Depletion and Alternative Promoter Usage,” Molecular Oncology 15, no. 8 (2021): 1995–2010. 33720519 10.1002/1878-0261.12944 PMC8334257

[13] R.‐C. Chai , Y.‐Z. Chang , X. Chang , et al., “YTHDF2 Facilitates UBXN1 mRNA Decay by Recognizing METTL3‐Mediated m6A Modification to Activate NF‐κB and Promote the Malignant Progression of Glioma,” Journal of Hematology & Oncology 14, no. 1 (2021): 109. 34246306 10.1186/s13045-021-01124-z PMC8272379

[14] Y. Yan , A. Luo , S. Liu , et al., “METTL3‐Mediated LINC00475 Alternative Splicing Promotes Glioma Progression by Inducing Mitochondrial Fission,” Research (Washington D C) 7 (2024): 0324. 38405130 10.34133/research.0324 PMC10886067

[15] J. He , L. Yuan , H. Lin , et al., “Genetic Variants in m6A Modification Core Genes Are Associated With Glioma Risk in Chinese Children,” Molecular Therapy—Oncolytics 20 (2021): 199–208. 33665358 10.1016/j.omto.2020.12.013 PMC7889446

[16] Y.‐P. Chen , Y.‐X. Liao , Z.‐J. Zhuo , et al., “Association Between Genetic Polymorphisms of Base Excision Repair Pathway and Glioma Susceptibility in Chinese Children,” World Journal of Pediatrics 18, no. 9 (2022): 632–635. 35543809 10.1007/s12519-022-00562-0

[17] Y. Wu , J. Zhou , J. Zhang , et al., “Pertinence of Glioma and Single Nucleotide Polymorphism of TERT, CCDC26, CDKN2A/B and RTEL1 Genes in Glioma: A Meta‐Analysis,” Frontiers in Oncology 13 (2023): 1180099. 37746290 10.3389/fonc.2023.1180099 PMC10512948

[18] C.‐H. Chen , K.‐C. Wei , W.‐C. Liao , et al., “Prognostic Value of an APOBEC3 Deletion Polymorphism for Glioma Patients in Taiwan,” Journal of Neurosurgery 138, no. 5 (2023): 1325–1337. 36152319 10.3171/2022.7.JNS2250

[19] T. Pienkowski , T. Kowalczyk , N. Garcia‐Romero , A. Ayuso‐Sacido , and M. Ciborowski , “Proteomics and Metabolomics Approach in Adult and Pediatric Glioma Diagnostics,” Biochimica Et Biophysica Acta. Reviews on Cancer 1877, no. 3 (2022): 188721. 35304294 10.1016/j.bbcan.2022.188721

[20] P. Aggarwal , W. Luo , K. C. Pehlivan , et al., “Pediatric Versus Adult High Grade Glioma: Immunotherapeutic and Genomic Considerations,” Frontiers in Immunology 13 (2022): 1038096. 36483545 10.3389/fimmu.2022.1038096 PMC9722734

[21] A. Kowalczyk , J. Zarychta , A. Marszołek , J. Zawitkowska , and M. Lejman , “Chimeric Antigen Receptor T Cell and Chimeric Antigen Receptor NK Cell Therapy in Pediatric and Adult High‐Grade Glioma‐Recent Advances,” Cancers (Basel) 16, no. 3 (2024): 623. 38339374 10.3390/cancers16030623 PMC10854514

[22] C.‐C. Chang and K.‐C. Hsia , “More Than a zip Code: Global Modulation of Cellular Function by Nuclear Localization Signals,” FEBS Journal 288, no. 19 (2021): 5569–5585. 33296547 10.1111/febs.15659

[23] M. El‐Tanani , H. Nsairat , V. Mishra , et al., “Ran GTPase and Its Importance in Cellular Signaling and Malignant Phenotype,” International Journal of Molecular Sciences 24, no. 4 (2023): 3065. 36834476 10.3390/ijms24043065 PMC9968026

[24] J. Czigleczki , P. T. de Resende Lara , B. Dudas , et al., “Small GTPase Ran: Depicting the Nucleotide‐Specific Conformational Landscape of the Functionally Important C‐Terminus,” Frontiers in Molecular Biosciences 10 (2023): 1111574. 36726377 10.3389/fmolb.2023.1111574 PMC9885160

[25] L. H. Ngo , A. G. Bert , B. K. Dredge , et al., “Nuclear Export of Circular RNA,” Nature 627, no. 8002 (2024): 212–220. 38355801 10.1038/s41586-024-07060-5

[26] C. Galiger , F. T. Zohora , C. Dorneburg , D. Tews , K.‐M. Debatin , and C. Beltinger , “The Survivin‐Ran Inhibitor LLP‐3 Decreases Oxidative Phosphorylation, Glycolysis and Growth of Neuroblastoma Cells,” BMC Cancer 23, no. 1 (2023): 1148. 38007466 10.1186/s12885-023-11635-2 PMC10676583

[27] C. Sheng , J. Qiu , Y. Wang , et al., “Knockdown of Ran GTPase Expression Inhibits the Proliferation and Migration of Breast Cancer Cells,” Molecular Medicine Reports 18, no. 1 (2018): 157–168. 29750309 10.3892/mmr.2018.8952 PMC6059664

[28] M. El‐Tanani , A. Platt‐Higgins , Y.‐F. Lee , et al., “Matrix Metalloproteinase 2 Is a Target of the RAN‐GTP Pathway and Mediates Migration, Invasion and Metastasis in Human Breast Cancer,” Life Sciences 310 (2022): 121046. 36209829 10.1016/j.lfs.2022.121046

[29] Y. Lu , Q. Meng , L. Bai , et al., “LINC00858 Stabilizes RAN Expression and Promotes Metastasis of Gastric Cancer,” Biology Direct 17, no. 1 (2022): 41. 36528654 10.1186/s13062-022-00355-5 PMC9759904

[30] C. Zhang , X. Zhao , W. Du , et al., “Ran Promotes the Proliferation and Migration Ability of Head and Neck Squamous Cell Carcinoma Cells,” Pathology, Research and Practice 216, no. 6 (2020): 152951. 32334891 10.1016/j.prp.2020.152951

[31] S. Elsheikh , I. Kouzoukakis , C. Fielden , et al., “Ran GTPase Is an Independent Prognostic Marker in Malignant Melanoma Which Promotes Tumour Cell Migration and Invasion,” Journal of Clinical Pathology 75, no. 1 (2022): 24–29. 33234696 10.1136/jclinpath-2020-206871

[32] X. Huang , J. Zhao , W. Fu , et al., “The Association of RAN and RANBP2 Gene Polymerphisms With Wilms Tumor Risk in Chinese Children,” Journal of Cancer 11, no. 4 (2020): 804–809. 31949483 10.7150/jca.36651 PMC6959007

[33] J. M. Levine , N. Ahsan , E. Ho , and J. D. Santoro , “Genetic Acute Necrotizing Encephalopathy Associated With RANBP2: Clinical and Therapeutic Implications in Pediatrics,” Multiple Sclerosis and Related Disorders 43 (2020): 102194. 32426208 10.1016/j.msard.2020.102194 PMC7228726

[34] X. Liu , J. Liu , W. Xiao , et al., “SIRT1 Regulates N^6^‐Methyladenosine RNA Modification in Hepatocarcinogenesis by Inducing RANBP_2_‐Dependent FTO SUMOylation,” Hepatology 72, no. 6 (2020): 2029–2050. 32154934 10.1002/hep.31222

[35] K. L. Sheng , K. J. Pridham , Z. Sheng , S. Lamouille , and R. T. Varghese , “Functional Blockade of Small GTPase RAN Inhibits Glioblastoma Cell Viability,” Frontiers in Oncology 8 (2018): 662. 30671385 10.3389/fonc.2018.00662 PMC6331428

[36] J. Hou , Y. Liu , P. Huang , et al., “RANBP10 Promotes Glioblastoma Progression by Regulating the FBXW7/c‐Myc Pathway,” Cell Death & Disease 12, no. 11 (2021): 967. 34671019 10.1038/s41419-021-04207-4 PMC8528885

[37] B. Oldrini , W.‐Y. Hsieh , H. Erdjument‐Bromage , et al., “EGFR Feedback‐Inhibition by Ran‐Binding Protein 6 Is Disrupted in Cancer,” Nature Communications 8, no. 1 (2017): 2035. 10.1038/s41467-017-02185-w PMC5725448 29229958

[38] Y.‐J. Kahm , I.‐G. Kim , and R.‐K. Kim , “RanBP1: A Potential Therapeutic Target for Cancer Stem Cells in Lung Cancer and Glioma,” International Journal of Molecular Sciences 24, no. 7 (2023): 6855. 37047826 10.3390/ijms24076855 PMC10095367

[39] A. A. Ayanlaja , G. Ji , J. Wang , et al., “Doublecortin Undergo Nucleocytoplasmic Transport via the RanGTPase Signaling to Promote Glioma Progression,” Cell Communication and Signaling: CCS 18, no. 1 (2020): 24. 32050972 10.1186/s12964-019-0485-5 PMC7017634

[40] T. Huang , Y. Yang , X. Song , et al., “PRMT6 Methylation of RCC1 Regulates Mitosis, Tumorigenicity, and Radiation Response of Glioblastoma Stem Cells,” Molecular Cell 81, no. 6 (2021): 1276–1291.e9. 33539787 10.1016/j.molcel.2021.01.015 PMC7979509

[41] I. S. Woo , H.‐S. Jang , S. Y. Eun , et al., “Ran Suppresses Paclitaxel‐Induced Apoptosis in Human Glioblastoma Cells,” Apoptosis 13, no. 10 (2008): 1223–1231. 18690538 10.1007/s10495-008-0247-0

[42] R. Schmits , B. Cochlovius , G. Treitz , et al., “Analysis of the Antibody Repertoire of Astrocytoma Patients Against Antigens Expressed by Gliomas,” International Journal of Cancer 98, no. 1 (2002): 73–77. 11857388 10.1002/ijc.10170

[43] V. Cimica , H.‐C. Chen , J. K. Iyer , and N. C. Reich , “Dynamics of the STAT3 Transcription Factor: Nuclear Import Dependent on Ran and Importin‐β1,” PLoS One 6, no. 5 (2011): e20188. 21625522 10.1371/journal.pone.0020188 PMC3098288

[44] J. E. Kim , M. Patel , J. Ruzevick , C. M. Jackson , and M. Lim , “STAT3 Activation in Glioblastoma: Biochemical and Therapeutic Implications,” Cancers (Basel) 6, no. 1 (2014): 376–395. 24518612 10.3390/cancers6010376 PMC3980601

[45] C. Piperi , K. A. Papavassiliou , and A. G. Papavassiliou , “Pivotal Role of STAT3 in Shaping Glioblastoma Immune Microenvironment,” Cells 8, no. 11 (2019): 1398. 31698775 10.3390/cells8111398 PMC6912524

